# Identification of the susceptible genes and mechanism underlying the comorbid presence of coronary artery disease and rheumatoid arthritis: a network modularization analysis

**DOI:** 10.1186/s12864-023-09519-7

**Published:** 2023-07-20

**Authors:** Siqi Zhang, Qikai Niu, Lin Tong, Sihong Liu, Pengqian Wang, Haiyu Xu, Bing Li, Huamin Zhang

**Affiliations:** 1 Institute of Chinese Materia Medica, China Academy of Chinese Medical Sciences, Beijing, 100700 China; 2 Institute of Information on Traditional Chinese Medicine, China Academy of Chinese Medical Sciences, Beijing, 100700 China

**Keywords:** Coronary artery disease, Rheumatoid arthritis, Comorbidity mechanism, Network module, Susceptibility gene

## Abstract

**Objective:**

The comorbidities of coronary artery disease (CAD) and rheumatoid arthritis (RA) are mutual risk factors, which lead to higher mortality, but the biological mechanisms connecting the two remain unclear. Here, we aimed to identify the risk genes for the comorbid presence of these two complex diseases using a network modularization approach, to offer insights into clinical therapy and drug development for these diseases.

**Method:**

The expression profile data of patients CAD with and without RA were obtained from the GEO database (GSE110008). Based on the differentially expressed genes (DEGs), weighted gene co-expression network analysis (WGCNA) was used to construct a gene network, detect co-expression modules, and explore their relation to clinical traits. The Z_summary_ index, gene significance (GS), and module membership (MM) were utilized to screen the important differentiated modules and hub genes. The GO and KEGG pathway enrichment analysis were applied to analyze potential mechanisms.

**Result:**

Based on the 278 DEGs obtained, 41 modules were identified, of which 17 and 24 modules were positively and negatively correlated with the comorbid occurrence of CAD and RA (CAD&RA), respectively. Thirteen modules with Z_summary_ < 2 were found to be the underlying modules, which may be related to CAD&RA. With GS ≥ 0.5 and MM ≥ 0.8, 49 hub genes were identified, such as *ADO, ABCA11P, POT1, ZNF141, GPATCH8, ATF6* and *MIA3*, etc. The area under the curve values of the representative seven hub genes under the three models (LR, KNN, SVM) were greater than 0.88. Enrichment analysis revealed that the biological functions of the targeted modules were mainly involved in cAMP-dependent protein kinase activity, demethylase activity, regulation of calcium ion import, positive regulation of tyrosine, phosphorylation of STAT protein, and tissue migration, etc.

**Conclusion:**

Thirteen characteristic modules and 49 susceptibility hub genes were identified, and their corresponding molecular functions may reflect the underlying mechanism of CAD&RA, hence providing insights into the development of clinical therapies against these diseases.

**Supplementary Information:**

The online version contains supplementary material available at 10.1186/s12864-023-09519-7.

## Introduction

The comorbidity of coronary artery disease and rheumatoid arthritis (CAD&RA) can lead to higher mortality rates than those of independent diseases [1], but the biological mechanisms connecting the two remain unclear. Patients with rheumatoid arthritis (RA) have a markedly higher incidence and mortality of cardiovascular disease than general population [2, 3]. Patients with RA accompanied with coronary artery disease (CAD) and revascularization by percutaneous coronary intervention (PCI) are significantly correlated with a higher risk of long-term, major adverse cardiac events [4]. Meanwhile, RA has been considered an independent risk factor for CAD development [5, 6]. Patients with RA have a larger coronary plaque and inflammation burden compared to patients without RA [7–9]. Some CAD-related risk factors, such as dyslipidemia, type 2 diabetes mellitus, hypertension, could also contribute to the CAD risk for patients with RA [10, 11]. Drugs such as corticosteroids, which are utilized for treating RA, might increase cardiovascular risk factors and aggravate heart diseases [12].

The underlying mechanisms of CAD-associated progression of RA are not fully elucidated. Studies found that the high mortality of CAD&RA is due to endothelial dysfunction and the circulating acute phase reactants such as C-reactive proteins [13, 14]. Inflammation can promote coronary atherosclerosis and induce coronary microvascular dysfunction in patients with RA, leading to an inadequate supply of myocardial oxygen, with the primary incipient procedures for the two changes being endothelial dysfunction and immune system dysregulation [15, 16]. Neutrophil activation-related genes of *S100A8* and *S100A12* are under investigation as therapeutic targets for both RA and CAD, hinting at the common pathogenic mechanisms of CAD&RA [17].

To discover the complex pathological mechanisms of CAD and RA, the conventional single target paradigm is not enough to illuminate the molecular basis of CAD&RA, and a novel systematic paradigm is urgently required. Rather than a simple view of the disease due to individual genomic variations, it requires network perspectives to understand the complex phenome-genome relationships of diseases and their comorbidities. Network medicine is thought to be capable of uncovering complex disease relationships using disease modules and network-based approaches, which may help to discover the shared biological mechanisms of associated diseases [18, 19]. A complex disease is rarely the direct consequence of a single gene alternation; rather, it is the result of the interaction of multiple molecular processes. Disease genes usually interact with each other and form closely connected subgraphs, i.e. disease modules, which play important roles in disease–disease relationships [20]. The identification of precise disease modules may help us understand the molecular interactions of complex diseases. Understanding comorbidities can also help physicians evaluate disease progression and improve treatment. Disease-related genes have been used to assess the similarity between different diseases [21, 22]. Accordingly, module-based strategies rather than single gene and targeted strategies are becoming increasingly important for revealing the relationship between multiple gene interactions and disease mechanisms [23, 24].

In this study, the gene expression array profile of CAD patients with and without RA was used to construct gene co-expression networks by weighted gene co-expression network analysis (WGCNA). Network modularization analyses were performed to identify the characteristic modules and susceptibility hub genes for CAD&RA to reveal the potential molecular mechanisms of the comorbid presence of CAD and RA. The workflow is shown in Fig. S1.

## Materials and methods

### Gene expression profile data and differentially expressed genes analysis

The CAD and CAD&RA datasets GSE110008 were downloaded from the National Center for Biotechnology Information (NCBI) Gene Expression Omnibus (GEO) (https://www.ncbi.nlm.nih.gov/geo/). The datasets included eight CAD&RA and eight control CAD samples according to the analysis of biopsies of the ascending aorta, and the platform was Affymetrix Human Genome U133A 2.0 Array (HG-U133A_2). The primary data was annotated to form an expression matrix, each probe was matched to their homologous gene symbols, and the repeated gene symbols in the matrix were excluded.

Differentially expressed genes (DEGs) between CAD&RA and CAD patients were identified using R (version 4.1.1) limma package. Genes with a false discovery rate (FDR) adjusted to *p* < 0.05 were considered as DEGs. Then, the DEGs were compared with the CAD-related and RA-related genes. To obtain CAD-related and RA-related genes, data were retrieved using the key words “coronary artery disease” and “rheumatoid arthritis” in the HPO (https://hpo.jax.org/app/), OMIM (https://omim.org/) and dbSNP (https://www.ncbi.nlm.nih.gov/snp/) databases during October, 2021.

### WGCNA network construction and clinical traits analysis

The R package WGCNA was applied to construct the DEG co-expression network. The DEG dataset was checked through the goodSamplesGenes step in WGCNA [25] to remove unqualified genes which do not qualify for inclusion because of missing values in multiple samples. The co-expression network of DEGs was constructed using appropriate soft-threshold β. Topological overlap measure (TOM) and Dynamic Hybrid Tree Cut algorithm were used to perform hierarchical clustering and partition the branches of dendrogram as a module with the following parameters (minModuleSize = 3, mergeCutHeight = 0.25 and verbose = 3). Then, the correlation coefficient between the expression level of each module and the different disease traits was analyzed.

### Analysis of module preservation using Z_summary_ statistic

To quantitatively analyze whether modules significantly varied between different disease groups, Z_summary_ [26] statistic was calculated to screen the differentiated modules between CAD and CAD&RA. Modules with a Z_summary_ ≥ 2 were regarded as preserved common modules, and if a module had a Z_summary_ score < 2, it was defined as a differentiated characteristic module for CAD&RA. Each identified modules was visualized by the Cytoscape software (version 3.7.2) to display the overall gene relationships that were obtained within a module [27].

### Functional enrichment analysis

Genes in the selected modules and all DEGs were respectively uploaded for functional enrichment analysis in the Metascape website (https://metascape.org/). The website is an open tool that helps the biomedical research community analyze gene/protein lists and make better data-driven decisions. A Gene Ontology (GO) function enrichment analysis and a Kyoto Encyclopedia of Genes and Genomes (KEGG) pathway enrichment analysis [28] were conducted to identify the function and pathways correlated with these modules (Count = 2; EASE = 0.01; and species = Homo sapiens). *P* < 0.05 was considered as the cutoff criterion. DiNGO [29] software was used to perform the HPO functional enrichment analysis.

### Identification of hub genes

The module membership (MM) was defined as the correlation between a gene and a given module [30]. At the same time, the gene significance (GS) of the gene in the module represented the correlation of the gene with clinical traits [31]. Genes with GS ≥ 0.5 and MM ≥ 0.8 in the clinically related gene module networks were defined as hub genes. Then, the expression level of hub genes with higher GS and MM rank were compared between the CAD and CAD&RA groups. A typical t-test was conducted to compare the difference in the expression with a *p* < 0.05 to indicate statistical significance. To validate whether these hub genes can classify patients into CAD or CAD&RA, three models—logistic regression (LR) [32], K-nearest neighbor (KNN) [33] and support vector machine (SVM) [34] were applied. Moreover, we used two other data-driven methods to screen featured genes for CAD&RA, i.e. the homogeneity of variance test in machine learning and Chi-square test (χ2) in statistics. Two other methods, i.e. Sab [20] and shortest distance [35], were used to calculate the proximity between the screened genes and CAD/RA-related disease genes from network angel. Smaller Sab and shortest distance values indicate closer proximity between the gene and diseases.

## Results

### DEGs between CAD and CAD&RA

**Fig. 1:**
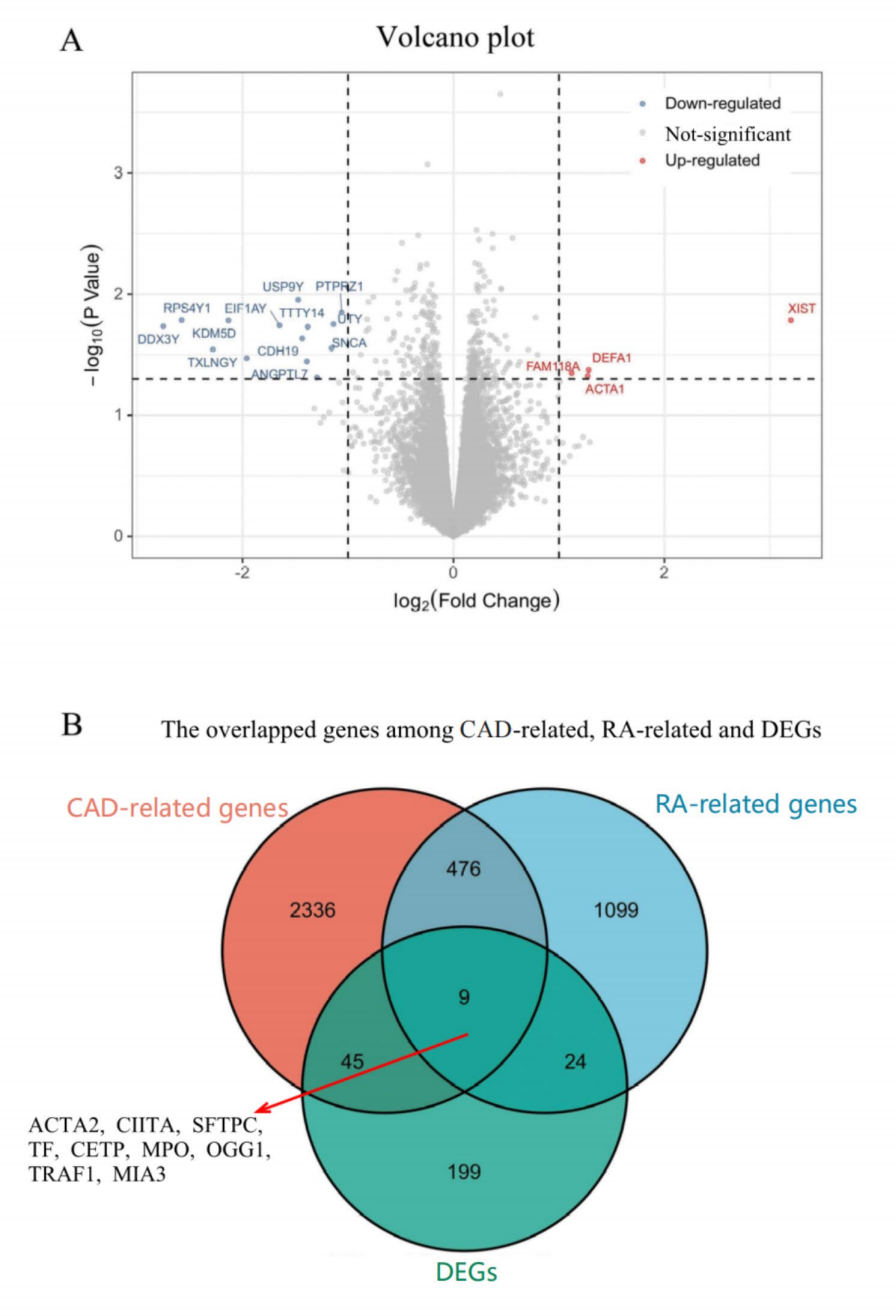
DEGs between CAD and CAD&RA. **(A)** Volcano plot for the gene’ expression level of GSE110008. Purple dots represent the down-regulated genes (log2Fold Change<-1, p < 0.05), red dots represent up-regulated genes (log2Fold Change > 1, p < 0.05). **(B)** The overlapped genes between the DEGs and known CAD, RA-related genes

Among all the 22,215 genes in GSE110008, 278 DEGs (Fig. 1A, Table. S1) were ultimately obtained after the duplicate genes were removed. In the up-regulated genes, *XIST, DEFA1, ACTA1*, and *FAM118A* genes’ differential expression were the most significant, and *DDX3Y, RPS4Y1, TXLNGY*, and *KDM5D* genes’ differential expression were the most significant among the down-regulated genes.

CAD and RA-related genes were obtained from the HPO, OMIM and dbSNP databases (Table. S2). Nine DEGs overlapped with both CAD and RA-related genes, and 54, 33 and 485 overlapped genes were detected between DEGs and CAD-related genes, DEGs and RA-related genes, CAD-related and RA-related genes, respectively (Fig. 1B).

### Co-expression modules identification and their correlation to clinical traits

**Fig. 2:**
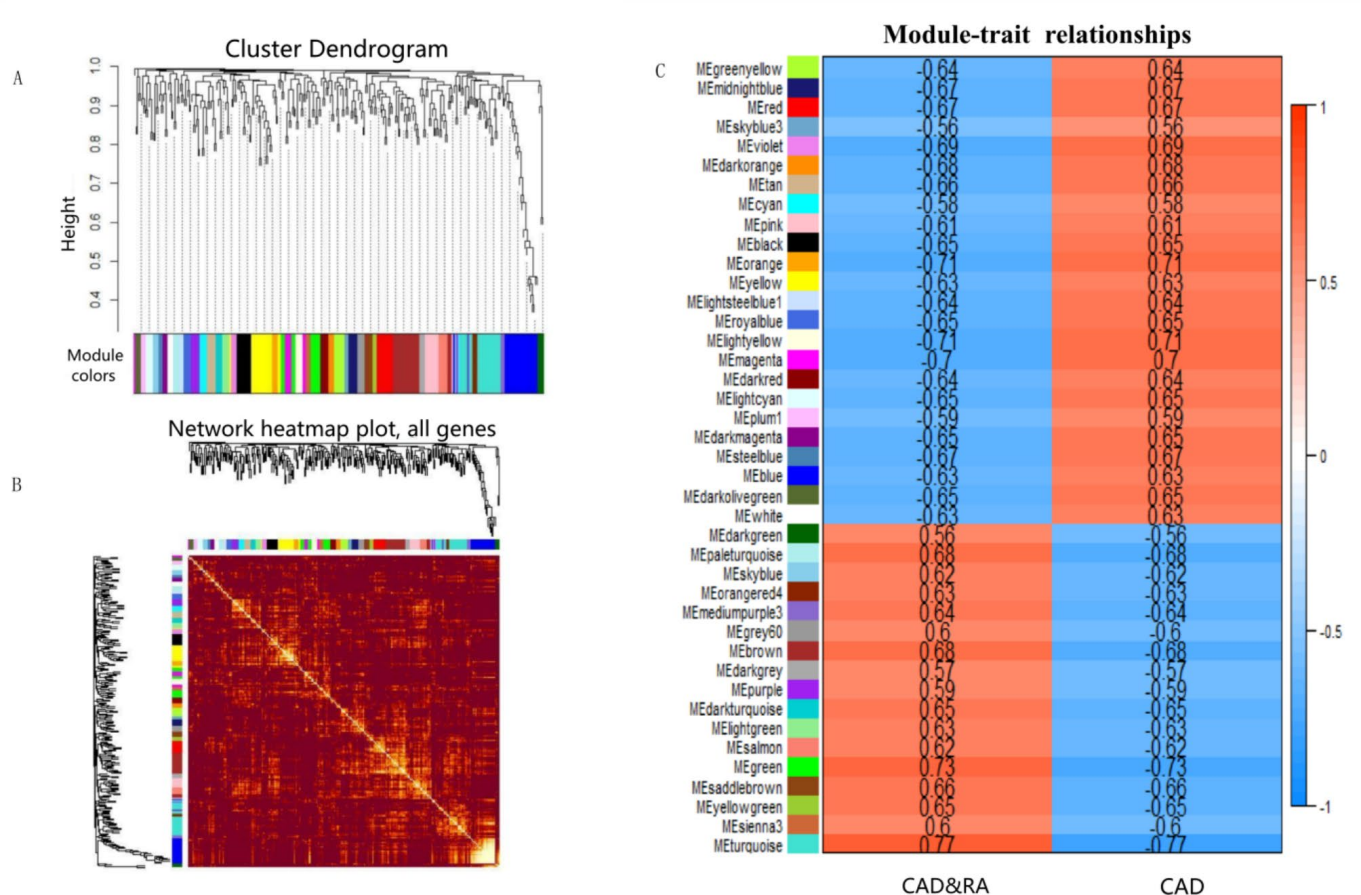
Identification of gene co-expression networks, modules and the correlation with clinical traits. **(A)** Cluster dendrogram of 278 DEGs based on the topological overlap. Each branch of the cluster tree with a certain color represents a co-expression module. **(B)** Heatmap of the topological overlap matrix (TOM) among all 41 modules of DEGs. **(C)** Heatmap of module-trait relationships. Each row represents a module, and each column represents a trait. Each cell contains the corresponding correlation coefficient

The one-step network construction function in WGCNA was used to construct the gene co-expression network based on the DEGs of CAD&RA. According to the scale-free independence and mean connectivity of the gene matrix, the soft thresholding power *β* was set at 7. Therefore, 41 modules were obtained, ranging in size from 3 ~ 24 (Table. S3). The cluster dendrogram of module distribution and the coexpression network heatmap are shown in (Fig. 2A-B).

To confirm the modules’ preservation and reproducibility, 1/2 and 3/4 of the samples were selected as testing sets and the Z_summary_ value [26] for each module was calculated. In the 1/2 sample testing set, 100% modules had a Z_summary_ score ≥ 0, 78.05% modules had a Z_summary_ score ≥ 2; In the 3/4 sample testing set, 100% modules with Z_summary_ score ≥ 0, and 92.68% modules with Z_summary_ score ≥ 2. These values demonstrate the robustness of our identified modules (Fig. S2).

To define the modules’ clinical characteristic, the correlation coefficient between modules’ expression and disease clinical traits was calculated. Overall, all the 41 modules, 35 modules (85.37%) had an absolute correlation coefficient to CAD or CAD&RA of over 0.6 (Fig. 2C). Among these modules, 17 modules were positively correlated with CAD&RA and negatively correlated with CAD. Conversely, 24 modules were positively correlated with CAD and negatively correlated with CAD&RA. Among the modules positively correlated with CAD&RA, the turquoise module had the largest corresponding correlation coefficient (0.77, *p* = 5 e − 04). For the modules negatively correlated with CAD&RA, the orange module (-0.71, *p* = 0.002), yellow module (-0.71, *p* = 0.002) and magenta module (-0.7, *p* = 0.002) had higher correlation coefficients (Fig. 2C).

### The characteristic differentiated modules of CAD&RA identification

**Fig. 3:**
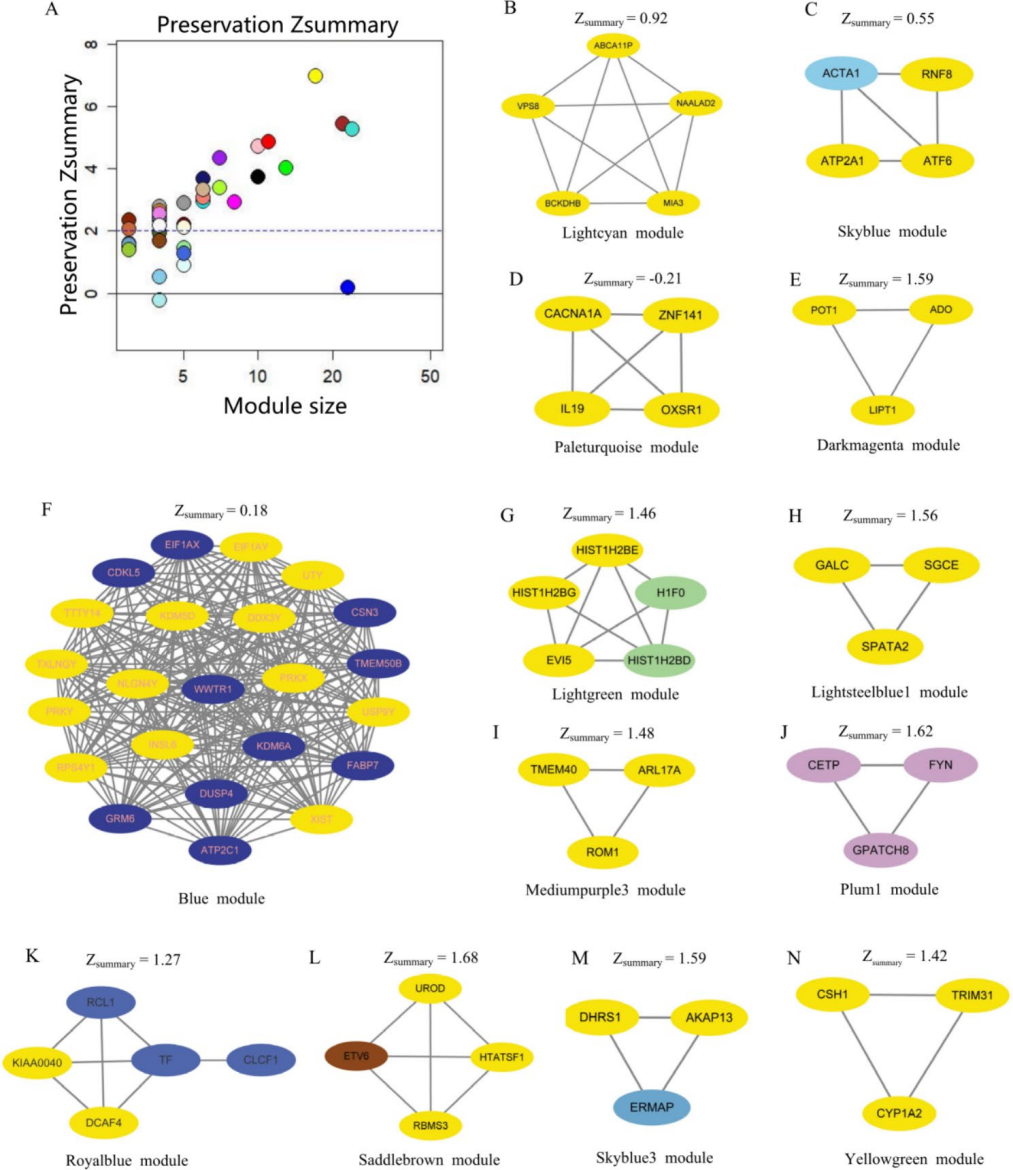
The identified differentiated modules for CAD&RA. **(A)** Preservation analysis of defined modules using Zsummary. The x-axis represents module size; the y-axis represents the Zsummary value. Each labeled color represents a module. The dashed blue line indicates the threshold Zsummary = 2. **(B-N)** Networks of the 13 characteristic modules. The genes marked in yellow are hub genes

Judging from the Z_summary_, the preserved modules (Z_summary_ ≥ 2) and differentiated modules (Z_summary_ < 2) for CAD&RA were identified (Fig. 3A). Finally, 13 modules were selected as the characteristic modules for CAD&RA, i.e., the blue, darkmagenta, lightcyan, lightgreen, lightsteelblue1, mediumpurple3, paleturquoise, plum1, royalblue, saddlebrown, skyblue, skyblue3 and yellowgreen module. In particular, the paleturquoise (*OXSR1, ZNF141, CACNA1A, IL19*) had a Z_summary_ less than 0 (Z_summary_ =-0.21), which represents the obvious differentiation between the two groups. All of the 13 selected modules are shown in Fig. 3(B-N). In addition to Z_summary_, the overall expression values of 13 differentially expressed modules between CAD&RA and CAD were assessed using a t-test. We found that six modules have significant differences between the two groups (Fig. S3).

### Functional and pathway enrichment analysis

**Fig. 4:**
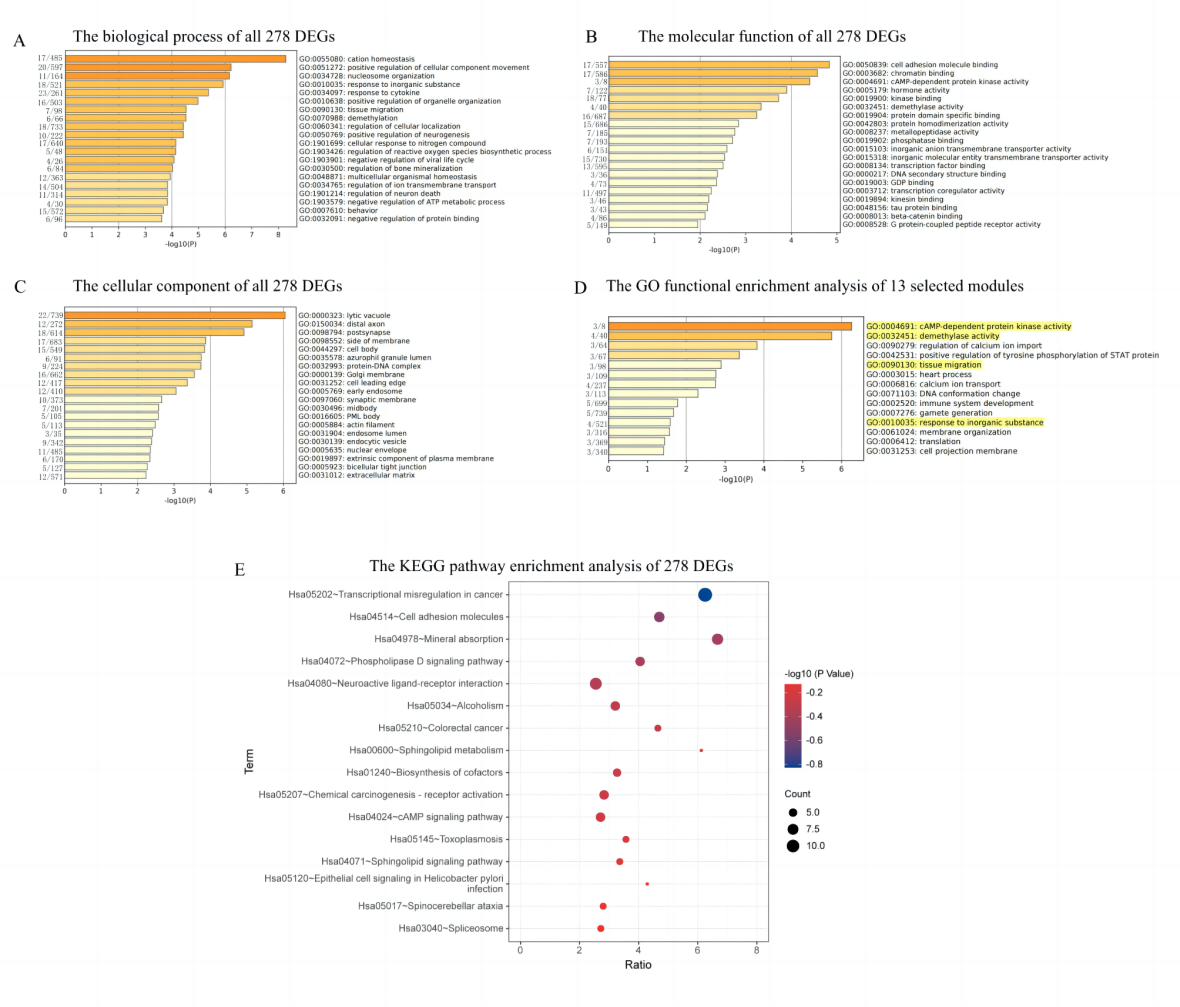
The enriched GO terms and KEGG pathways. **(A-C)** Biological process, molecular function, cellular component in GO function for all 278 DEGs. **(D)** Enriched GO functions of 13 selected modules. On the left of each figure are the on-target numbers of the enriched genes in certain GO terms. **(E)** Enriched KEGG pathways of 278 DEGs

GO function enrichment analysis and KEGG pathway enrichment analysis were performed in 13 differentiated modules and all DEGs respectively. In the GO functional enrichment analysis of all 278 DEGs, the top 20 terms were selected by the *p* value in each category. Thus, for biological processes (Fig. 4A), the genes were mainly enriched in cation homeostasis, positive regulation of cellular component movement, and nucleosome organization. Regarding the molecular functions (Fig. 4B), the genes were mainly enriched in cell adhesion molecule binding, chromatin binding and cAMP-dependent protein kinase activity. When it comes to cellular components (Fig. 4C), the genes were mainly enriched in lytic vacuole, distal axon and postsynapse. For the 13 differentiated modules, there were 14 enriched GO functions (Fig. 4D), which were mainly cAMP-dependent protein kinase activity, demethylase activity, and regulation of calcium ion import.

For the top 20 GO terms, some are common to multiple modules, and some are unique. For example, cAMP-dependent protein kinase activity and heart process are common terms of the blue and skyblue modules; the positive regulation of tyrosine phosphorylation of STAT protein and response to inorganic substance are common terms of yellow, plum3 and royalblue modules. In addition, the term of translation is unique to the blue module (Table. S4).

Two overlapped functions were found between the biological process of DEGs and the 13 modules, tissue migration and response to inorganic substances. Similarly, 2 enriched overlapping molecular functions of DEGs and 13 modules were found, which were cAMP-dependent protein kinase activity and demethylase activity.

In terms of KEGG pathway enrichment analysis, we found that the DEGs could enrich multiple pathways (Fig. 4E). The top three pathways were transcriptional misregulation in cancer, cell adhesion molecules, and mineral absorption. Furthermore, HPO functional enrichment analysis revealed that 13 differentially expressed modules were significantly enriched to 2 HPO terms. One of these terms was Y-linked inheritance and the other was gonosomal inheritance, which contains the genes: *DDX3Y*, *KDM5D*, *CDKL5*, *USP9Y*, *ROM1*, *KDM6A.* Moreover, among the 16 enriched pathways, 12 genes were enriched in transcriptional misregulation in cancer, accounting for 4.4%; 9 genes were enriched in the neuroactive ligand-receptor interaction pathway, accounting for 3.3%; and 8 genes were enriched in cell adhesion molecules pathway, accounting for 2.93% (Table. S5).

### The identified hub genes for CAD&RA

**Fig. 5:**
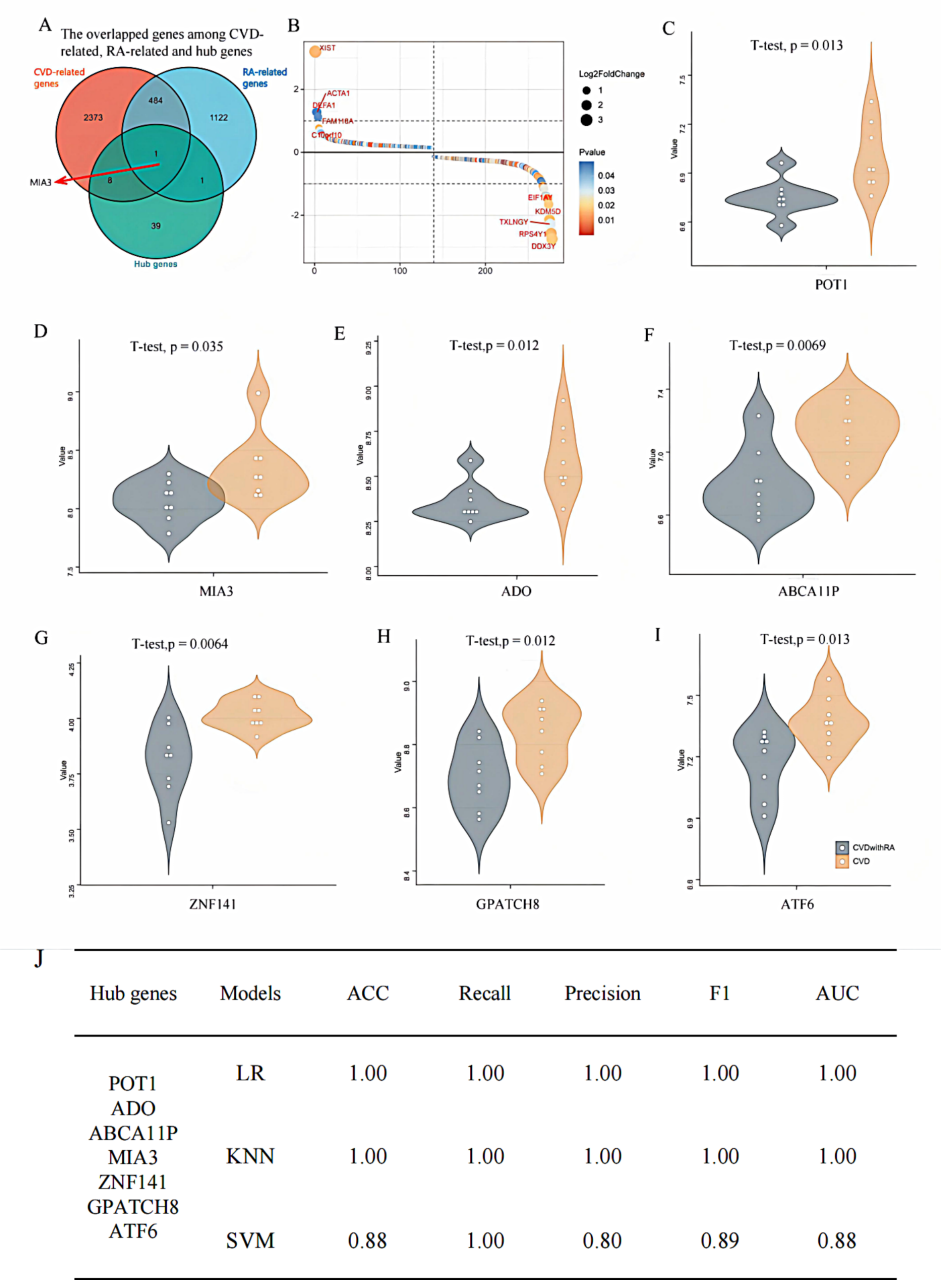
**(A)** The overlapped genes among hub gene and known CAD, RA-related genes. **(B)** Gene expression difference significance ranking. X-axis represents the rank of DEGs, Y-axis represents log2FoldChange. **(C-I)** The expression level of representative hub genes between CAD and CAD&RA. **(J)** Classification ability of the three models based on the representative seven hub genes

The 13 selected modules contained 68 genes. With a GS over 0.5 and a MM over 0.8 as cut-off criteria, 49 genes were identified as hub genes (Table 1). Seven out of these 49 hub genes had a GS greater than 0.6 and MM greater than 0.9, i.e., *POT1, ADO, ABCA11P*, *GALC, ZNF141, GPATCH8* and *ATF6*. Compared to the known CAD and RA-related genes, 9, 2 and 485 genes were found between CAD-related and hub genes, RA-related and hub genes, CAD-related and RA-related genes, respectively (Fig. 5A). Interestingly, the *MIA3* overlapped hub genes were found to be related to both CAD and RA.

Based on their significance, the top five up-regulated genes were *XIST, DEFA1*, *ACTA1, FAM118A* and *C10orf10*, in which the *XIST* was also a hub gene. The top five down-regulated genes were *EIF1AY, KDM5D, TXLNGY, RPS4Y1* and *DDX3Y*, and all of them were hub genes. The expression level of representative hub genes was significantly different between CAD and CAD&RA (Fig. 5C-I). The results showed that *ZNF141* was down-regulated in CAD&RA, while other genes were up-regulated.

**Table 1:** Hub genes of the differentiated modules for CAD&RA (GS＞0.5 and MM＞0.8)

Module	Gene	GS	MM
Blue	RPS4Y1	-0.55	0.96
	PRKX	-0.58	0.83
	EIF1AY	-0.54	0.98
	DDX3Y	-0.54	0.96
	PRKY	-0.58	0.91
	USP9Y	-0.58	0.96
	KDM5D	-0.55	0.98
	TTTY14	-0.54	0.98
	NLGN4Y	-0.55	0.89
	UTY	-0.55	0.98
	TXLNGY	-0.51	0.95
	INSL6	0.53	-0.82
	XIST	0.55	-0.91
Darkmagenta	**POT1**	**-0.63**	**0.96**
	LIPT1	-0.54	0.88
	**ADO**	**-0.63**	**0.93**
Lightcyan	VPS8	-0.56	0.89
	MIA3	-0.54	0.83
	BCKDHB	-0.51	0.87
	**ABCA11P**	**-0.65**	**0.9**
	NAALAD2	-0.54	0.85
Lightsteelblue1	**GALC**	**-0.6**	**0.95**
	SPATA2	-0.61	0.86
	SGCE	-0.52	0.88
Mediumpurple3	ROM1	0.63	0.88
	ARL17A	-0.55	-0.87
	TMEM40	0.55	0.94
Paleturquoise	OXSR1	0.57	0.85
	**ZNF141**	**-0.68**	**-0.91**
	CACNA1A	0.58	0.87
	IL19	0.56	0.87
Plum1	**GPATCH8**	**-0.61**	**0.92**
	FYN	-0.5	0.92
Royalblue	KIAA0040	0.54	-0.87
	DCAF4	-0.59	0.84
Saddlebrown	HTATSF1	0.59	0.91
	RBMS3	0.63	0.87
	UROD	-0.59	-0.94
Skyblue	RNF8	-0.56	-0.8
	ATP2A1	0.52	0.84
	**ATF6**	**-0.6**	**-0.92**
Skyblue3	DHRS1	-0.5	0.86
	AKAP13	0.51	-0.95
Yellowgreen	CSH1	0.55	0.9
	CYP1A2	0.58	0.95
	TRIM31	0.67	0.89
Lightgreen	HIST1H2BG	0.58	0.87
	HIST1H2BE	0.64	0.84
	EVI5	-0.54	-0.93

Moreover, the area under the curve (AUC) values of the 7 hub genes under the three models were greater than 0.88 (Fig. 5J). Compared with the top 7 featured genes based on the homogeneity of variance test (*TMX1*, *TCF7L2*, *CDC6*, *ZNF157*, *HIST3H3*, *COQ7* and *CLDN18*) and χ2 test (*XIST*, *DDX3Y*, *TXLNGY*, *RPS4Y1*, *KDM5D*, *USP9Y* and *EIF1AY*), our 7 susceptible genes (*POT1*, *ADO*, *ABCA11P*, *MIA3*, *ZNF141*, *GPATCH8* and *ATF6*) based on GS/MM yielded optimized results in the three models, with AUC of 1.00, 1.00 and 0.88 for LR, KNN, and SVM, respectively (Table. S6), which indicated the excellent classification effect of 7 hub genes. In addition, our 7 hub genes had smaller Sab and shortest distance values than those of genes identified by other two methods, indicating the superiority of the susceptible genes identified by our modular-based analysis (Table S7).

## Discussion

Considering the increased mortality for the comorbid presence of CAD and RA [1–3], it is essential to uncover the underlying mechanisms of CAD&RA. For the complexity of CAD and RA, a network modularization approach was used to identify the characteristic module and susceptibility gene for CAD&RA. Thus, 13 modules and 49 hub genes that were related with CAD&RA were screened, and further functional enrichment analysis revealed their potential mechanisms.

Among the identified hub genes, several were reportedly related to CAD or RA. A study demonstrated that the *IL19* risk allele was relevant to stroke/MI in SLE and RA, but not in the general population, showing that shared immune pathways may be contained in cardiovascular disease pathogenesis and inflammatory rheumatic diseases [36]. The expression of *UTY* and *PRKY* was found associated with the risk of CAD [37, 38]. Studies have proved that the SNP rs17465637 in the *MIA3* gene was associated with the risk of CAD and RA [39, 40]. Another study found that high-intensity interval training (HIIT) could improve RA skeletal muscle gene-*BCKDHB*, which can increase amino acid catabolism and interconversion [41]. *FYN* is one of genes that is likely to play a significant role in maintenance and functioning of several of the replicated pathways of CAD [42]. Simultaneously, *FYN* gene is a diagnostic biomarker and one of key driver genes in RA synovial tissue subtypes C1 and C3 [43–45]. The *ATF6* gene also plays an important role in both CAD and RA [46, 47]. Additionally, a study analyzed that *AKAP13* was one the of hub genes unique to CAD [48], a finding consistent with our study. Another study showed that *CYP1A2* genotype can modify the risk of RA and CYP1A2*1F allele may relate to leflunomide toxicity in RA patients therapy [49, 50]. A previous study has shown that *INSL6* which produced by TNF-polarized macrophages can stimulate bone formation in mice with RA [51]. In addition, the binding of *XIST* to GATA1 can promote to RA [52]. The genes *LIPT1* [53, 54] and *TMEM40*[55] were reported to be susceptibility genes with RA. Moreover, *POT1* expression levels are significantly lower in RA than in the control group in vitro [56]. Mass spectrometry results revealed [57] that *GALC* expression levels were significantly increased in patients with atherosclerosis. In samples collected from male patients with new-onset heart failure, the *RPS4Y1* was overexpression [58]. In addition, coffee intake is correlated with a risk of nonfatal myocardial infarction; this correlation is believed to be influenced by *CYP1A2*, which is related with the development of RA in Korea [59, 60]. After infarction, the expression of *CACNA1A* can enhance cardiac differentiation of brown adipose-derived stem cells to regenerate the myocardium after infarction [61]. Besides, using advanced technologies of lncRNA subcellular localization and silencing, *lnc-KDM5D-4* expression was shown to be associated with atherosclerosis and CAD in men [62].

A total of 14 GO function terms were enriched by the differentiated modules, the top three terms were cAMP-dependent protein kinase activity, demethylase activity, and regulation of calcium ion import. A related study found that during cardiac preservation, a cAMP pulse could reduce the incidence and severity of transplant-related CAD [63]. Vasoactive intestinal peptide (VIP) may be an effective anti-RA treatment because it leads to the elevation of intracellular cAMP, which can inhibit TNF-α production in macrophages [64]. Another study indicates that a combination of cilostazol and MTX can activate the cAMP-dependent protein kinase pathway in the synovial fibroblasts resulting in the suppression of the inflammation of RA [65]. Fibroblast-like synoviocytes (FLSs) are involved in RA joint destruction, and pathologic process and elevated JMJD3 promotes the proliferation and migration of FLS [66]. A study found that the` Janus kinase-signal transducer and activator of transcription (JAK-STAT) pathway is an emerging target in inflammation, mainly in RA, and it heightens the cardiovascular risk [67]. Overexpression of a histone demethylase KDM4B could boost cell growth, migration and invasion, and inhibit apoptosis of FLS in RA by activating STAT3 signaling [68]. Basal intracellular calcium ion concentrations in patients with inactive RA were significantly higher than in healthy individuals, which in turn were greater than in the active RA group, which showed the important roles of calcium ions in the pathological process of RA [69].

In the top three pathways of 16 KEGG pathways, cell adhesion molecules and mineral absorption were associated with both CAD and RA. For the pathway related to cell adhesion molecules, the expression levels of cell adhesion molecules increased in patients with RA, and were associated with disease activity, oxidative stress, and inflammatory markers targeting the expression of these molecules is an important therapeutic strategy for RA [70, 71]. Moreover, the expression of both CDC42 and microRNA-34a was correlated with that of cell adhesion molecules in patients with CAD [72, 73]. For the mineral absorption pathway, clinical trials revealed that the concentrations of the mineral copper were higher in patients with RA than in healthy people [74], and zinc and selenium levels in patients with CAD admitted for coronary artery bypass grafting were reduced compared to those before surgery [75].

Although we have found several of the related modules and susceptible genes, certain limitations for our study also exist. For limited datasets and samples involving the comorbidities, CAD and RA, cross validation could not be performed. With further clinical sequencing and updated cardiovascular disease and RA-related databases, investigations should continue to validate the modular mechanism of CAD&RA. Besides, the proposed susceptible genes also need further experimental and clinical validation.

In conclusion, thirteen characteristic modules and 49 susceptible hub genes for CAD&RA were identified by network modularization analysis, including *ADO*, *ABCA11P, GALC, ZNF141, GPATCH8, ATF6, MIA3*, etc. These hub genes and their corresponding molecular functions may reflect the underlying mechanism of CAD&RA, which can provide novel perspectives for their clinical therapy strategies and precise drug discovery.

## Electronic supplementary material

Below is the link to the electronic supplementary material.

Supplementary Material 1: **Table. S1** 278 differentially expressed genes.

Supplementary Material 2: **Table. S2** CAD-related and RA-related genes in 3 disease databases.

Supplementary Material 3: **Table. S3** The relationship between gene and module.

Supplementary Material 4: **Table. S4** The common and unique GO items between modules.

Supplementary Material 5: **Table. S5** The KEGG pathway of 278 DEGs.

Supplementary Material 6: **Table. S6** The comparison of 7 susceptible genes with other featured genes.

Supplementary Material 7: **Table. S7** The proximity between genes and CAD&RA-related diseases genes

Supplementary Material 8: Overall workflow of this study. **Fig. S1A** The source of gene expression profile data of CAD and CAD&RA and differentially expressed genes analysis. **B** The overlapped genes among CAD, RA and DEGs in disease databases. **C** Co-expression network construction, module division and clinical traits analysis. **D** The identification of differentiated modules using Zsummary statistic and hub genes screening. **E** Functional and pathway enrichment analysis, gene expression difference significance ranking analysis and the expression level of representive hub genes

Supplementary Material 9: **Fig. S2** Validation the robustness of modules with Zsummary. The x-axis represents module size; the y-axis represents the Zsummary value. Each labeled color represents a module. The dashed blue line indicates the threshold Zsummary = 2. **A** 1/2 samples of CAD and CAD&RA test. **B** 3/4 samples of CAD and CAD&RA test.

Supplementary Material 10: **Fig. S3** Evaluation the 13 differentiated modules with t-test. The x-axis represents module; the y-axis represents the p value.

Supplementary Material 11: The code of this study.

## Data Availability

The raw data of GSE110008 are freely available from GEO database.

## References

[1] Wang H, Li X, Gong G (2020). Cardiovascular outcomes in patients with co-existing coronary artery disease and rheumatoid arthritis: a systematic review and meta-analysis. Med (Baltim).

[2] Meune C, Touzé E, Trinquart L, Allanore Y (2009). Trends in cardiovascular mortality in patients with rheumatoid arthritis over 50 years: a systematic review and meta-analysis of cohort studies. Rheumatology (Oxford).

[3] Avina-Zubieta JA, Thomas J, Sadatsafavi M, Lehman AJ, Lacaille D (2012). Risk of incident cardiovascular events in patients with rheumatoid arthritis: a meta-analysis of observational studies. Ann Rheum Dis.

[4] Spartera M, Godino C, Baldissera E (2017). Long-term clinical outcomes of patients with rheumatoid arthritis and concomitant coronary artery disease. Am J Cardiovasc Dis.

[5] Kitas GD, Erb N (2003). Tackling ischaemic heart disease in rheumatoid arthritis. Rheumatology (Oxford).

[6] Solomon DH, Karlson EW, Rimm EB (2003). Cardiovascular morbidity and mortality in women diagnosed with rheumatoid arthritis. Circulation.

[7] Karpouzas GA, Malpeso J, Choi TY, Li D, Munoz S, Budoff MJ (2014). Prevalence, extent and composition of coronary plaque in patients with rheumatoid arthritis without symptoms or prior diagnosis of coronary artery disease. Ann Rheum Dis.

[8] Aubry MC, Maradit-Kremers H, Reinalda MS, Crowson CS, Edwards WD, Gabriel SE (2007). Differences in atherosclerotic coronary heart disease between subjects with and without rheumatoid arthritis. J Rheumatol.

[9] Skeoch S, Bruce IN (2015). Atherosclerosis in rheumatoid arthritis: is it all about inflammation?. Nat Rev Rheumatol.

[10] Kremers HM, Crowson CS, Therneau TM, Roger VL, Gabriel SE (2008). High ten-year risk of cardiovascular disease in newly diagnosed rheumatoid arthritis patients: a population-based cohort study. Arthritis Rheum.

[11] Solomon DH, Curhan GC, Rimm EB, Cannuscio CC, Karlson EW (2004). Cardiovascular risk factors in women with and without rheumatoid arthritis. Arthritis Rheum.

[12] Hafström I, Rohani M, Deneberg S, Wörnert M, Jogestrand T, Frostegård J (2007). Effects of low-dose prednisolone on endothelial function, atherosclerosis, and traditional risk factors for atherosclerosis in patients with rheumatoid arthritis–a randomized study. J Rheumatol.

[13] Mudau M, Genis A, Lochner A, Strijdom H (2012). Endothelial dysfunction: the early predictor of atherosclerosis. Cardiovasc J Afr.

[14] Crowson CS, Liao KP, Davis JM (2013). Rheumatoid arthritis and cardiovascular disease. Am Heart J.

[15] Faccini A, Kaski JC, Camici PG (2016). Coronary microvascular dysfunction in chronic inflammatory rheumatoid diseases. Eur Heart J.

[16] Wang L, Zhang Y, Zhang SY (2019). Immunotherapy for the rheumatoid arthritis-associated coronary artery disease: promise and future. Chin Med J (Engl).

[17] Jessee R, Peart E, Beineke P (2017). Rheumatoid arthritis complicates noninvasive whole blood gene expression testing for coronary artery disease. Am Heart J.

[18] Barabási AL, Gulbahce N, Loscalzo J (2011). Network medicine: a network-based approach to human disease. Nat Rev Genet.

[19] Bloom JS, Kotenko I, Sadhu MJ, Treusch S, Albert FW, Kruglyak L (2015). Genetic interactions contribute less than additive effects to quantitative trait variation in yeast. Nat Commun.

[20] Menche J, Sharma A, Kitsak M (2015). Disease networks. Uncovering disease-disease relationships through the incomplete interactome. Science.

[21] Sarfati D, Koczwara B, Jackson C (2016). The impact of comorbidity on cancer and its treatment. CA Cancer J Clin.

[22] Goh KI, Cusick ME, Valle D, Childs B, Vidal M, Barabási AL (2007). The human disease network. Proc Natl Acad Sci U S A.

[23] Beltrao P, Cagney G, Krogan NJ (2010). Quantitative genetic interactions reveal biological modularity. Cell.

[24] Sales-Pardo M (2017). The importance of being modular. Science.

[25] Langfelder P, Horvath S (2008). WGCNA: an R package for weighted correlation network analysis. BMC Bioinformatics.

[26] Langfelder P, Luo R, Oldham MC, Horvath S. Is my Network Module Preserved and Reproducible? Plos Comput Biol 2011;7. 10.1371/journal.pcbi.1001057 PMC3024255 21283776

[27] Shannon P, Markiel A, Ozier O (2003). Cytoscape: a software environment for integrated models of biomolecular interaction networks. Genome Res.

[28] Kanehisa M, Furumichi M, Sato Y, Kawashima M, Ishiguro-Watanabe M (2023). KEGG for taxonomy-based analysis of pathways and genomes. Nucleic Acids Res.

[29] Davidović R, Perovic V, Gemovic B, Veljkovic N (2019).

[30] Ren Y, van Blitterswijk M, Allen M (2018). TMEM106B haplotypes have distinct gene expression patterns in aged brain. Mol Neurodegener.

[31] Yang Q, Wang R, Wei B et al. Candidate Biomarkers and Molecular Mechanism Investigation for Glioblastoma Multiforme Utilizing WGCNA. Biomed Res Int. 2018;2018:4246703. Published 2018 Sep 26. 10.1155/2018/4246703. 10.1155/2018/4246703 PMC6178162 30356407

[32] Dreiseitl S, Ohno-Machado L (2002). Logistic regression and artificial neural network classification models: a methodology review. J Biomed Inform.

[33] Zhang S, Li X, Zong M, Zhu X, Wang R (2018). Efficient kNN classification with different numbers of nearest neighbors. IEEE Trans Neural Netw Learn Syst.

[34] Keerthi SS, Shevade SK, Bhattacharyya C (2001). Murthy; improvements to Platt’s SMO algorithm for SVM Classifier Design. Neural Comput.

[35] Wang Y, Yang H, Chen L, Jafari M, Tang J (2021). Network-based modeling of herb combinations in traditional chinese medicine. Brief Bioinform.

[36] Leonard D, Svenungsson E, Dahlqvist J (2018). Novel gene variants associated with cardiovascular disease in systemic lupus erythematosus and rheumatoid arthritis. Ann Rheum Dis.

[37] Bloomer LD, Nelson CP, Eales J (2013). Male-specific region of the Y chromosome and cardiovascular risk: phylogenetic analysis and gene expression studies. Arterioscler Thromb Vasc Biol.

[38] Eales JM, Maan AA, Xu X (2019). Human Y chromosome exerts Pleiotropic Effects on susceptibility to atherosclerosis. Arterioscler Thromb Vasc Biol.

[39] Koch W, Schatke A, Wolferstetter H, Mueller JC, Schömig A, Kastrati A (2011). Extended evidence for association between the melanoma inhibitory activity 3 gene and myocardial infarction. Thromb Haemost.

[40] García-Bermúdez M, López-Mejías R, González-Juanatey C (2012). Association study of MIA3 rs17465637 polymorphism with cardiovascular disease in rheumatoid arthritis patients. DNA Cell Biol.

[41] Andonian BJ, Johannemann A, Hubal MJ (2021). Altered skeletal muscle metabolic pathways, age, systemic inflammation, and low cardiorespiratory fitness associate with improvements in disease activity following high-intensity interval training in persons with rheumatoid arthritis. Arthritis Res Ther.

[42] Ghosh S, Vivar J, Nelson CP (2015). Systems Genetics analysis of genome-wide Association Study reveals Novel Associations between key biological processes and coronary artery disease. Arterioscler Thromb Vasc Biol.

[43] Liu J, Chen N (2021). A 9 mRNAs-based diagnostic signature for rheumatoid arthritis by integrating bioinformatic analysis and machine-learning. J Orthop Surg Res.

[44] Jung SM, Park KS, Kim KJ (2021). Deep phenotyping of synovial molecular signatures by integrative systems analysis in rheumatoid arthritis. Rheumatology (Oxford).

[45] Brisslert M, Bian L, Svensson MN (2014). S100A4 regulates the src-tyrosine kinase dependent differentiation of Th17 cells in rheumatoid arthritis. Biochim Biophys Acta.

[46] Gonzales K, Feng V, Bikkina P, Landicho MA, Haas MJ, Mooradian AD (2021). The effect of nicotine and dextrose on endoplasmic reticulum stress in human coronary artery endothelial cells. Toxicol Res (Camb).

[47] Lee WS, Jeong JH, Lee EG (2018). Tacrolimus regulates endoplasmic reticulum stress-mediated osteoclastogenesis and inflammation: in vitro and collagen-induced arthritis mouse model. Cell Biol Int.

[48] Dong C, Tang L, Liu Z (2014). Landscape of the relationship between type 2 diabetes and coronary heart disease through an integrated gene network analysis. Gene.

[49] Cornelis MC, Bae SC, Kim I, El-Sohemy A (2010). CYP1A2 genotype and rheumatoid arthritis in Koreans. Rheumatol Int.

[50] Bohanec Grabar P, Rozman B, Tomsic M, Suput D, Logar D, Dolzan V (2008). Genetic polymorphism of CYP1A2 and the toxicity of leflunomide treatment in rheumatoid arthritis patients. Eur J Clin Pharmacol.

[51] Yi X, Liu X, Kenney HM (2021). TNF-Polarized macrophages produce insulin-like 6 peptide to stimulate bone formation in rheumatoid arthritis in mice. J Bone Miner Res.

[52] Yu B, Chen Y, Chen E (2023). LncRNA RNA XIST binding to GATA1 contributes to rheumatoid arthritis through its effects on proliferation of synovial fibroblasts and angiogenesis via regulation of CCN6. Mol Immunol.

[53] Zhou Y, Li X, Ng L (2023). Identification of copper death-associated molecular clusters and immunological profiles in rheumatoid arthritis. Front Immunol.

[54] Wang W, Chen Z, Hua Y. Bioinformatics Prediction and Experimental Validation Identify a Novel Cuproptosis-Related Gene Signature in Human Synovial Inflammation during Osteoarthritis Progression. Biomolecules. 2023;13(1):127. Published 2023 Jan 7. doi:10.3390/biom13010127. 10.3390/biom13010127 PMC9855951 36671512

[55] Yu X, Teng H, Marques A, Ashgari F, Ibrahim SM (2009). High resolution mapping of Cia3: a common arthritis quantitative trait loci in different species. J Immunol.

[56] Qing YF, Zhou JG, Zhao MC (2012). Altered expression of TPP1 in fibroblast-like synovial cells might be involved in the pathogenesis of rheumatoid arthritis. Rheumatol Int.

[57] Li M, Tong X, Lv P, Feng B, Yang L, Wu Z, Cui X, Bai Y, Huang Y, Liu H. A not-stop-flow online normal-/reversed-phase two-dimensional liquid chromatography-quadrupole time-of-flight mass spectrometry method for comprehensive lipid profiling of human plasma from atherosclerosis patients. J Chromatogr A. 2014 Dec 12;1372 C:110–119. doi: 10.1016/j.chroma.2014.10.094. Epub 2014 Nov 3. PMID: 25465009. 10.1016/j.chroma.2014.10.094 25465009

[58] Heidecker B, Lamirault G, Kasper EK, Wittstein IS, Champion HC, Breton E, Russell SD, Hall J, Kittleson MM, Baughman KL, Hare JM. The gene expression profile of patients with new-onset heart failure reveals important gender-specific differences. Eur Heart J. 2010 May;31(10):1188–96. 10.1093/eurheartj/ehp549. Epub 2009 Dec 22. PMID: 20031959; PMCID: PMC2869442. 10.1093/eurheartj/ehp549 PMC2869442 20031959

[59] Cornelis MC, El-Sohemy A, Kabagambe EK, Campos H, Coffee (2006). CYP1A2 genotype, and risk of myocardial infarction. JAMA.

[60] Cornelis MC, Bae SC, Kim I, El-Sohemy A (2010). CYP1A2 genotype and rheumatoid arthritis in Koreans. Rheumatol Int.

[61] Wang H, Shi J, Wang Y (2014). Promotion of cardiac differentiation of brown adipose derived stem cells by chitosan hydrogel for repair after myocardial infarction. Biomaterials.

[62] Molina E, Chew GS, Myers SA et al. A Novel Y-Specific Long Non-Coding RNA Associated with Cellular Lipid Accumulation in HepG2 cells and Atherosclerosis-related Genes. Sci Rep. 2017;7(1):16710. Published 2017 Dec 1. doi:10.1038/s41598-017-17165-9. 10.1038/s41598-017-17165-9 PMC5711902 29196750

[63] Wang CY, Aronson I, Takuma S (2000). cAMP pulse during preservation inhibits the late development of cardiac isograft and allograft vasculopathy. Circ Res.

[64] Foey AD, Field S, Ahmed S (2003). Impact of VIP and cAMP on the regulation of TNF-alpha and IL-10 production: implications for rheumatoid arthritis. Arthritis Res Ther.

[65] Kim HY, Lee SW, Park SY (2012). Efficacy of concurrent administration of cilostazol and methotrexate in rheumatoid arthritis: pharmacologic and clinical significance. Life Sci.

[66] Jia W, Wu W, Yang D (2018). Histone demethylase JMJD3 regulates fibroblast-like synoviocyte-mediated proliferation and joint destruction in rheumatoid arthritis. FASEB J.

[67] Baldini C, Moriconi FR, Galimberti S, Libby P, De Caterina R (2021). The JAK-STAT pathway: an emerging target for cardiovascular disease in rheumatoid arthritis and myeloproliferative neoplasms. Eur Heart J.

[68] Zhang X, Nan H, Guo J, Liu J (2021). KDM4B overexpression promotes the growth, Migration, and Invasion of Rheumatoid Arthritis Fibroblast-Like Synoviocytes by activating STAT3 pathway. Biochem Genet.

[69] Goulding NJ, Guyre PM (1992). Impairment of neutrophil fc gamma receptor mediated transmembrane signalling in active rheumatoid arthritis. Ann Rheum Dis.

[70] Salem HR, Zahran ES (2021). Vascular cell adhesion molecule-1 in rheumatoid arthritis patients: relation to disease activity, oxidative stress, and systemic inflammation. Saudi Med J.

[71] Veale DJ, Maple C (1996). Cell adhesion molecules in rheumatoid arthritis. Drugs Aging.

[72] Zhou M, Wu J, Tan G (2022). The relation of circulating cell division cycle 42 expression with Th1, Th2, and Th17 cells, adhesion molecules, and biochemical indexes in coronary heart disease patients. Ir J Med Sci.

[73] Li H, Chen M, Feng Q (2022). MicroRNA-34a in coronary heart disease: correlation with disease risk, blood lipid, stenosis degree, inflammatory cytokines, and cell adhesion molecules. J Clin Lab Anal.

[74] Yazar M, Sarban S, Kocyigit A, Isikan UE (2005). Synovial fluid and plasma selenium, copper, zinc, and iron concentrations in patients with rheumatoid arthritis and osteoarthritis. Biol Trace Elem Res.

[75] Al-Bader A, Christenson JT, Simonet F, Abul H, Dashti H, Schmuziger M (1998). Inflammatory response and oligo-element alterations following cardiopulmonary bypass in patients undergoing coronary artery bypass grafting. Cardiovasc Surg.

