# Supplementary figures and images for: Identification of the susceptible genes and mechanism underlying the comorbid presence of coronary artery disease and rheumatoid arthritis: a network modularization analysis
Source: BMC Genomics. 2023 Jul 20;24:411. doi: 10.1186/s12864-023-09519-7 (PMC10360345; doi:10.1186/s12864-023-09519-7)

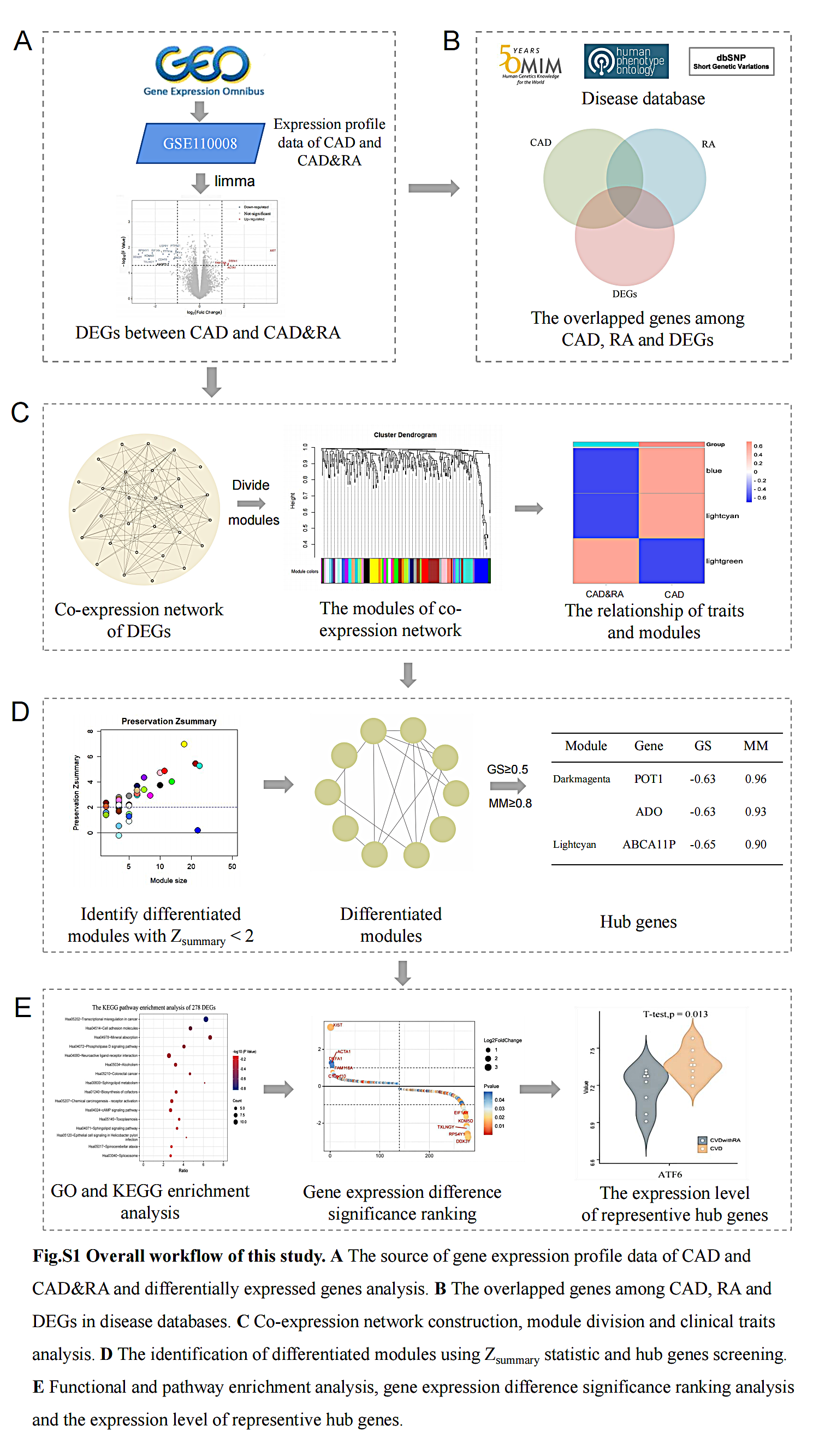

Supplement: Supplementary file 8 — Supplementary Material 8: Overall workflow of this study. Fig. S1A The source of gene expression profile data of CAD and CAD&RA and differentially expressed genes analysis. B The overlapped genes among CAD, RA and DEGs in disease databases. C Co-expression network construction, module division and clinical traits analysis. D The identification of differentiated modules using Zsummary statistic and hub genes screening. E Functional and pathway enrichment analysis, gene expression difference significance ranking analysis and the expression level of representive hub genes [file 12864_2023_9519_MOESM8_ESM.png]

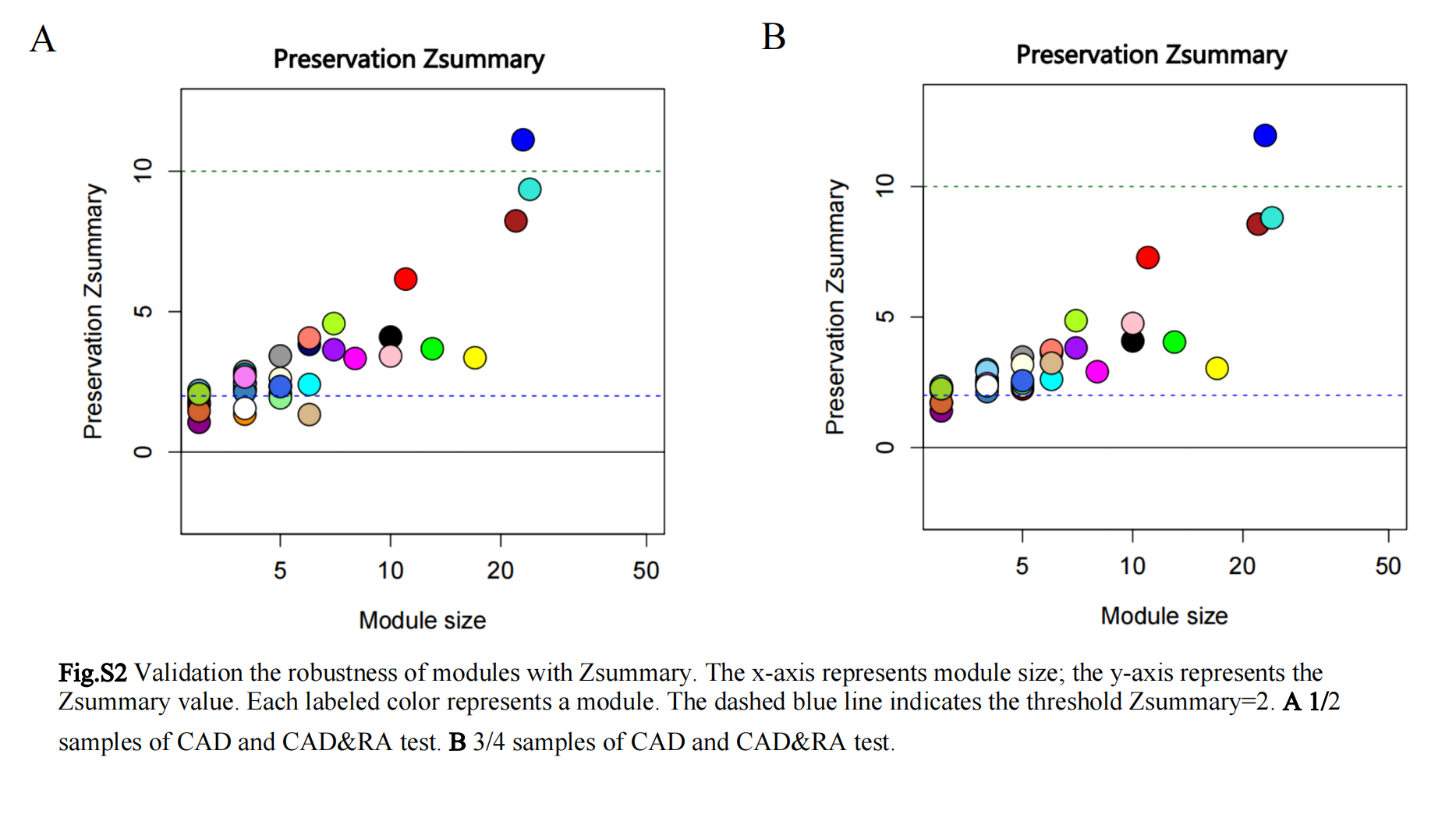

Supplement: Supplementary file 9 — Supplementary Material 9: Fig. S2 Validation the robustness of modules with Zsummary. The x-axis represents module size; the y-axis represents the Zsummary value. Each labeled color represents a module. The dashed blue line indicates the threshold Zsummary = 2. A 1/2 samples of CAD and CAD&RA test. B 3/4 samples of CAD and CAD&RA test. [file 12864_2023_9519_MOESM9_ESM.tif]

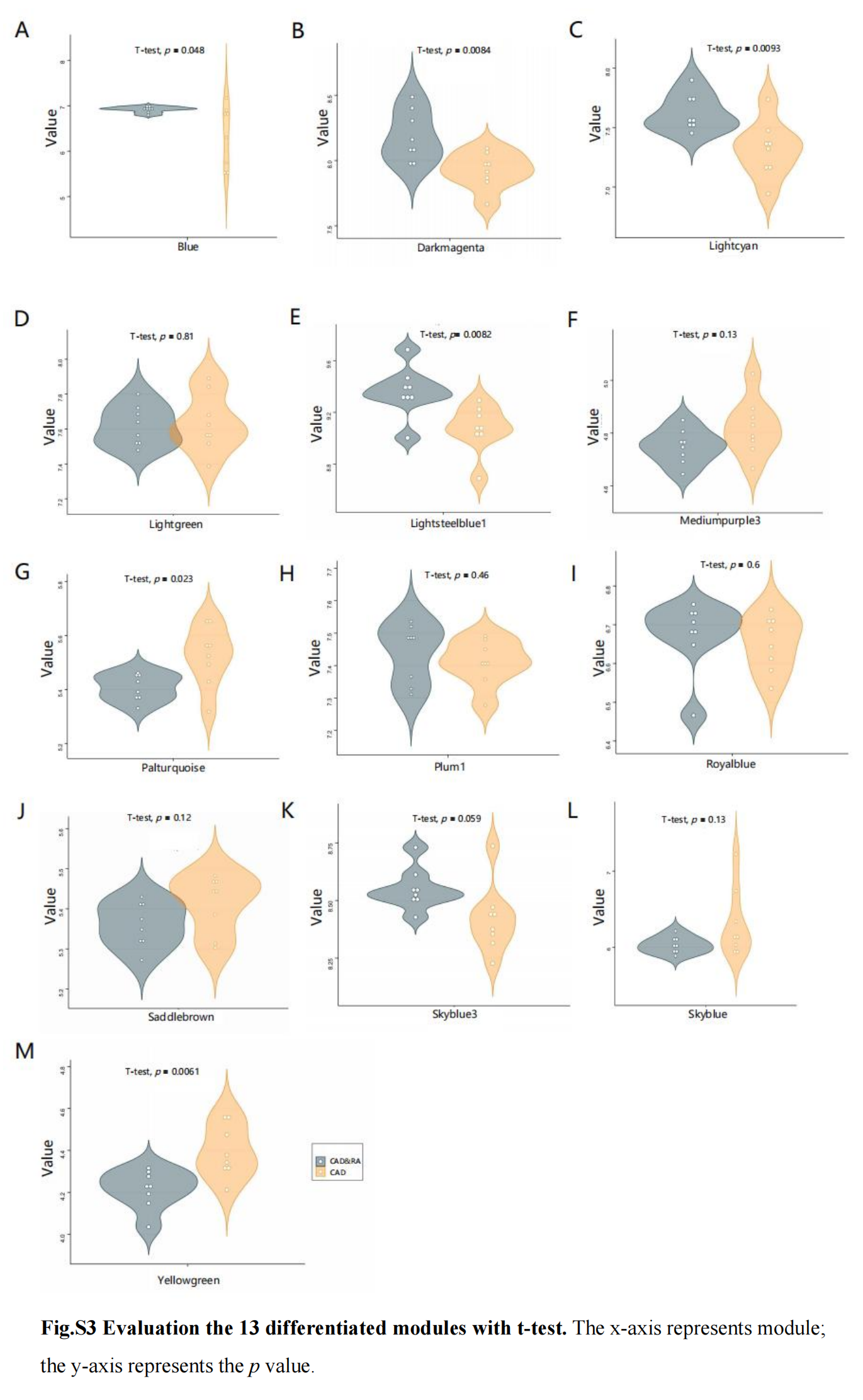

Supplement: Supplementary file 10 — Supplementary Material 10: Fig. S3 Evaluation the 13 differentiated modules with t-test. The x-axis represents module; the y-axis represents the p value. [file 12864_2023_9519_MOESM10_ESM.tif]
