# Supplementary material for: Identification of the susceptible genes and mechanism underlying the comorbid presence of coronary artery disease and rheumatoid arthritis: a network modularization analysis
Source: BMC Genomics. 2023 Jul 20;24:411. doi: 10.1186/s12864-023-09519-7 (PMC10360345; doi:10.1186/s12864-023-09519-7)
Supplement: Supplementary file 11 — Supplementary Material 11: The code of this study. [file 12864_2023_9519_MOESM11_ESM.docx]

WGCNA learning tutorial details can refer to the following website: https://horvath.genetics.ucla.edu/html/CoexpressionNetwork/Rpackages/WGCNA/

getwd();

# If necessary, change the path below to the directory where the data files are stored.

# "." means current directory. On Windows use a forward slash / instead of the usual \.

workingDir = "C:/Users/zsq/Desktop/after delete";

setwd(workingDir);

# Load the package

library(WGCNA);

# The following setting is important, do not omit.

options(stringsAsFactors = FALSE);

#Read in the CYJYs set

CYJYsData = read.csv("CYJYs.csv");

# Take a quick look at what is in the data set:

dim(CYJYsData);

names(CYJYsData);

datExpr = as.data.frame(t(CYJYsData[, -c(1)]));

names(datExpr) = CYJYsData$GENE;

rownames(datExpr) = names(CYJYsData)[-c(1)];

gsg = goodSamplesGenes(datExpr, verbose = 3);

gsg$allOK

#Write the data check results to the file

write.csv(gsg$goodGenes,file = "goodgene-a0-negative.csv");

#Save the dataset

save(datExpr, file = "CYJYs-a30negative-dataInput.RData")

#Compute the appropriate scale-free parameters

powers = c(c(1:10), seq(from = 12, to=20, by=2))

# Call the network topology analysis function

sft = pickSoftThreshold(datExpr, powerVector = powers, verbose = 5, blockSize = 20000)

# Plot the results:

sizeGrWindow(9, 5)

par(mfrow = c(1,2));

cex1 = 0.8;

# Scale-free topology fit index as a function of the soft-thresholding power

plot(sft$fitIndices[,1], -sign(sft$fitIndices[,3])*sft$fitIndices[,2],

xlab="Soft Threshold (power)",ylab="Scale Free Topology Model Fit,signed R^2",type="n",

main = paste("Scale independence"));

text(sft$fitIndices[,1], -sign(sft$fitIndices[,3])*sft$fitIndices[,2],

labels=powers,cex=cex1,col="red");

# this line corresponds to using an R^2 cut-off of h

abline(h=0.90,col="red")

# Mean connectivity as a function of the soft-thresholding power

plot(sft$fitIndices[,1], sft$fitIndices[,5],

xlab="Soft Threshold (power)",ylab="Mean Connectivity", type="n",

main = paste("Mean connectivity"))

text(sft$fitIndices[,1], sft$fitIndices[,5], labels=powers, cex=cex1,col="red")

##Build the network and divide module (pay attention to adjust the corresponding parameters)

net = blockwiseModules(datExpr, power = 7, maxBlockSize = 20000,

TOMType = "unsigned", minModuleSize = 3,

reassignThreshold = 0, mergeCutHeight = 0.25,

numericLabels = TRUE, pamRespectsDendro = FALSE,

saveTOMs = TRUE,

saveTOMFileBase = "CYJYsTOM",

verbose = 3)

##Draw network clustering diagram

sizeGrWindow(12, 9)

# Convert labels to colors for plotting

mergedColors = labels2colors(net$colors)

# Plot the dendrogram and the module colors underneath

plotDendroAndColors(net$dendrograms[[1]], mergedColors[net$blockGenes[[1]]],

"Module colors",

dendroLabels = FALSE, hang = 0.03,

addGuide = TRUE, guideHang = 0.05)

##Save the network- -module data

moduleLabels = net$colors

moduleColors = labels2colors(net$colors)

MEs = net$MEs;

geneTree = net$dendrograms[[1]];

save(MEs, moduleLabels, moduleColors, geneTree,

file = "CYJYs-networkConstruction-auto.RData")

table(net$colors)

write.csv(moduleLabels,file = "labels-CYJYsgroup.csv");

write.csv(moduleColors,file = "module-CYJYsgroup.csv");

##Draw the whole network cluster heat map (note that the power is consistent with before)

dissTOM = 1-TOMsimilarityFromExpr(datExpr, power = **7**);

# Transform dissTOM with a power to make moderately strong connections more visible in the heatmap

plotTOM = dissTOM^8;

# Set diagonal to NA for a nicer plot

diag(plotTOM) = NA;

# Call the plot function

sizeGrWindow(9,9)

TOMplot(plotTOM, geneTree, moduleColors, main = "Network heatmap plot, all genes")

##Comparison between the two data groups (e. g., disease, control group), and calculate differability of modules.

For Zsummary value, greater than 2 is conservative module, and less than 0 is differential module

# Read case group data（The module division results need to be added to the last column）

dat0=read.csv("DH-a-30-ref.csv", header=TRUE)

names(dat0)

#According to the data adjustment, the first column is the ID, and the last column is the module

datSummary=dat0[,c(1,20)]

#The column of expression matrix

datExprFemale <- t(dat0[,2:19])

no.samples <- dim(datExprFemale)[[1]]

dim(datExprFemale)

# Set the columns names to probe names and the title name of the first column

colnames(datExprFemale) = datSummary$Name

# This module assignment was obtained by libing

#The title name of the module column

colorsFemale = dat0$module

# Read the control group data

data=read.csv("DH-b-30-good.csv", header=TRUE)

#The column of expression matrix

datExprMale = t(data[,2:20])

colnames(datExprMale) = data$Name

dim(datExprMale)

setLabels = c("Female", "Male");

multiExpr = list(Female = list(data = datExprFemale), Male = list(data = datExprMale));

multiColor = list(Female = colorsFemale);

# Comparison of two groups (calculated Zsummary-value)

system.time( {

mp = modulePreservation(multiExpr, multiColor,

referenceNetworks = 1,

nPermutations = 200,

maxGoldModuleSize = 500,

maxModuleSize = 2000,

randomSeed = 1,

quickCor = 0,

verbose = 3)

} );

save(mp, file = "modulepreservation-case-control.RData");

# Output results（Zsummary、medianRank）

ref = 1

test = 2

statsObs = cbind(mp$quality$observed[[ref]][[test]][, -1], mp$preservation$observed[[ref]][[test]][, -1])

statsZ = cbind(mp$quality$Z[[ref]][[test]][, -1], mp$preservation$Z[[ref]][[test]][, -1]);

print( cbind(statsObs[, c("medianRank.pres", "medianRank.qual")],

signif(statsZ[, c("Zsummary.pres", "Zsummary.qual")], 2)) )

modColors = rownames(mp$preservation$observed[[ref]][[test]])

moduleSizes = mp$preservation$Z[[ref]][[test]][, 1];

# leave grey and gold modules out

plotMods = !(modColors %in% c("grey", "gold"));

# Text labels for points

text = modColors[0];

# Auxiliary convenience variable

plotData = cbind(mp$preservation$observed[[ref]][[test]][, 2], mp$preservation$Z[[ref]][[test]][, 2])

# Main titles for the plot

mains = c("Preservation Median rank", "Preservation Zsummary");

# Start the plot

sizeGrWindow(10, 5);

#pdf(fi="Plots/BxHLiverFemaleOnly-modulePreservation-Zsummary-medianRank.pdf", wi=10, h=5)

par(mfrow = c(1,2))

par(mar = c(4.5,4.5,2.5,1))

for (p in 1:2)

{

min = min(plotData[, p], na.rm = TRUE);

max = max(plotData[, p], na.rm = TRUE);

# Adjust ploting ranges appropriately

if (p==2)

{

if (min > -max/10) min = -max/10

ylim = c(min - 0.1 * (max-min), max + 0.1 * (max-min))

} else

ylim = c(max + 0.1 * (max-min), min - 0.1 * (max-min))

plot(moduleSizes[plotMods], plotData[plotMods, p], col = 1, bg = modColors[plotMods], pch = 21,

main = mains[p],

cex = 2.4,

ylab = mains[p], xlab = "Module size", log = "x",

ylim = ylim,

xlim = c(3, 10000), cex.lab = 1.2, cex.axis = 1.2, cex.main =1.4)

labelPoints(moduleSizes[plotMods], plotData[plotMods, p], text, cex = 1, offs = 0.08);

# For Zsummary, add threshold lines

if (p==2)

{

abline(h=0)

abline(h=2, col = "blue", lty = 2)

abline(h=10, col = "darkgreen", lty = 2)

}

}

write.csv(mp$preservation$Z,file = "Zvalue-case-control.csv");

write.csv(mp$preservation$log.p,file = "Zvalue-case-control-p-value.csv");

# Screen hub genes (one per module), power value was consistent with the previous ones

Hubgene = chooseOneHubInEachModule(datExpr, moduleColors, numGenes = 100, omitColors = "grey", power = 4, type = "signed")

write.csv(Hubgene, file = "Hubgene.csv");

# Screen hub genes (multiple per module), power values was consistent with the previous ones

Hubgenes = chooseTopHubInEachModule(datExpr, moduleColors,omitColors = "grey", power = 4, type = "signed")

write.csv(Hubgenes, file = "Hubgenes.csv");

# Output the network (or a module) to the cytoscape：

#Power values was consistent with the previous ones

TOM = TOMsimilarityFromExpr(datExpr, power = 5);

save(TOM, file = "TOM-data");

#Adjust the content according to the sample data format

annot = read.csv(file = "GeneAnnotation.csv");

# Select modules

modules = c("salmon");

probes = names(datExpr)

inModule = is.finite(match(moduleColors, modules));

modProbes = probes[inModule];

#The title name of the corresponding column in the read annot file

modGenes = annot$$gene_symbol[match(modProbes, annot$Name)];

# Select the corresponding Topological Overlap

modTOM = TOM[inModule, inModule];

dimnames(modTOM) = list(modProbes, modProbes)

# Export the network into edge and node list files Cytoscape can read

cyt = exportNetworkToCytoscape(modTOM,

edgeFile = paste("CytoscapeInput-edges-", paste(modules, collapse="-"), ".txt", sep=""),

nodeFile = paste("CytoscapeInput-nodes-", paste(modules, collapse="-"), ".txt", sep=""),

weighted = TRUE,

threshold = 0.02,

nodeNames = modProbes,

altNodeNames = modGenes,

nodeAttr = moduleColors[inModule]);

# Associated with the clinical data

# Read the clinical data

#Adjust the content according to the sample data format

traitData = read.csv("Trait.csv");

dim(traitData)

names(traitData)

allTraits = traitData[, -c(31, 16,20)];

#The first column is clinical features of concern, the latter columns are sample name column, consistent with the sample name listed in the expression data table

allTraits = allTraits[, c(2,3:4)];

dim(allTraits)

names(allTraits)

Samples = rownames(datExpr);

traitRows = match(Samples, allTraits$sample);

datTraits = allTraits[traitRows, -1];

rownames(datTraits) = allTraits$sample;

collectGarbage();

library(flashClust);

sampleTree2 = flashClust(dist(datExpr), method = "average")

traitColors = numbers2colors(datTraits, signed = FALSE);

sampleTree = flashClust(dist(datExpr), method = "average");

sizeGrWindow(12,9)

#pdf(file = "Plots/sampleClustering.pdf", width = 12, height = 9);

par(cex = 0.6);

par(mar = c(0,4,2,0))

plot(sampleTree, main = "Sample clustering to detect outliers", sub="", xlab="", cex.lab = 1.5,

cex.axis = 1.5, cex.main = 2)

# Define numbers of genes and samples

nGenes = ncol(datExpr);

nSamples = nrow(datExpr);

# Recalculate MEs with color labels

MEs0 = moduleEigengenes(datExpr, moduleColors)$eigengenes

MEs = orderMEs(MEs0)

moduleTraitCor = cor(MEs, datTraits, use = "p");

moduleTraitPvalue = corPvalueStudent(moduleTraitCor, nSamples);

write.csv(moduleTraitCor,file = "module-traits-Cor.csv");

write.csv(moduleTraitPvalue,file = "module-traits-pvalue.csv");

# Draw

sizeGrWindow(10,6)

# Will display correlations and their p-values

textMatrix = paste(signif(moduleTraitCor, 2), "\n(", signif(moduleTraitPvalue, 1), ")", sep = "");

dim(textMatrix) = dim(moduleTraitCor)

par(mar = c(6, 8.5, 3, 3));

# Display the correlation values within a heatmap plot

labeledHeatmap(Matrix = moduleTraitCor,

xLabels = names(datTraits),

yLabels = names(MEs),

ySymbols = names(MEs),

colorLabels = FALSE,

colors = blueWhiteRed(50),

textMatrix = textMatrix,

setStdMargins = FALSE,

cex.text = 0.5,

zlim = c(-1,1),

main = paste("Module-trait relationships"))

#Column of clinical features of concern-title name

weight = as.data.frame(datTraits$CADwithRA);

names(weight) = "CADwithRA"

# names (colors) of the modules

modNames = substring(names(MEs), 3)

geneModuleMembership = as.data.frame(cor(datExpr, MEs, use = "p"));

MMPvalue = as.data.frame(corPvalueStudent(as.matrix(geneModuleMembership), nSamples));

names(geneModuleMembership) = paste("MM", modNames, sep="");

names(MMPvalue) = paste("p.MM", modNames, sep="");

geneTraitSignificance = as.data.frame(cor(datExpr, weight, use = "p"));

GSPvalue = as.data.frame(corPvalueStudent(as.matrix(geneTraitSignificance), nSamples));

names(geneTraitSignificance) = paste("GS.", names(weight), sep="");

names(GSPvalue) = paste("p.GS.", names(weight), sep="");

write.csv(geneModuleMembership,file = "module-MM.csv");

write.csv(geneTraitSignificance,file = "module-GS.csv");

#Module of concern

module = "yellowgreen"

column = match(module, modNames);

moduleGenes = moduleColors==module;

sizeGrWindow(7, 7);

par(mfrow = c(1,1));

verboseScatterplot(abs(geneModuleMembership[moduleGenes, column]),

abs(geneTraitSignificance[moduleGenes, 1]),

xlab = paste("Module Membership in", module, "module"),

ylab = "Gene significance for TCM syndrome score",

main = paste("Module membership vs. gene significance\n"),

cex.main = 1.2, cex.lab = 1.2, cex.axis = 1.2, col = module)
